# Methane-Fueled Syntrophy through Extracellular Electron Transfer: Uncovering the Genomic Traits Conserved within Diverse Bacterial Partners of Anaerobic Methanotrophic Archaea

**DOI:** 10.1128/mBio.00530-17

**Published:** 2017-08-01

**Authors:** Connor T. Skennerton, Karuna Chourey, Ramsunder Iyer, Robert L. Hettich, Gene W. Tyson, Victoria J. Orphan

**Affiliations:** a Division of Geological and Planetary Sciences, California Institute of Technology, Pasadena, California, USA; b Chemical Sciences Division, Oak Ridge National Laboratory, Oak Ridge, Tennessee, USA; c Genome Science and Technology, University of Tennessee, Knoxville, Tennessee, USA; d Australian Centre for Ecogenomics, School of Chemistry and Molecular Biosciences, University of Queensland, Brisbane, Queensland, Australia; Max Planck Institute for Marine Microbiology

**Keywords:** ANME, AOM, anaerobic oxidation of methane, extracellular electron transfer, SEEP-SRB1, methane seeps, multiheme cytochrome, sulfate-reducing bacteria

## Abstract

The anaerobic oxidation of methane by anaerobic methanotrophic (ANME) archaea in syntrophic partnership with deltaproteobacterial sulfate-reducing bacteria (SRB) is the primary mechanism for methane removal in ocean sediments. The mechanism of their syntrophy has been the subject of much research as traditional intermediate compounds, such as hydrogen and formate, failed to decouple the partners. Recent findings have indicated the potential for extracellular electron transfer from ANME archaea to SRB, though it is unclear how extracellular electrons are integrated into the metabolism of the SRB partner. We used metagenomics to reconstruct eight genomes from the globally distributed SEEP-SRB1 clade of ANME partner bacteria to determine what genomic features are required for syntrophy. The SEEP-SRB1 genomes contain large multiheme cytochromes that were not found in previously described free-living SRB and also lack periplasmic hydrogenases that may prevent an independent lifestyle without an extracellular source of electrons from ANME archaea. Metaproteomics revealed the expression of these cytochromes at *in situ* methane seep sediments from three sites along the Pacific coast of the United States. Phylogenetic analysis showed that these cytochromes appear to have been horizontally transferred from metal-respiring members of the *Deltaproteobacteria* such as *Geobacter* and may allow these syntrophic SRB to accept extracellular electrons in place of other chemical/organic electron donors.

## IMPORTANCE

Some archaea, known as anaerobic methanotrophs, are capable of converting methane into carbon dioxide when they are growing syntopically with sulfate-reducing bacteria. This partnership is the primary mechanism for methane removal in ocean sediments; however, there is still much to learn about how this syntrophy works. Previous studies have failed to identify the metabolic intermediate, such as hydrogen or formate, that is passed between partners. However, recent analysis of methanotrophic archaea has suggested that the syntrophy is formed through direct electron transfer. In this research, we analyzed the genomes of multiple partner bacteria and showed that they also contain the genes necessary to perform extracellular electron transfer, which are absent in related bacteria that do not form syntrophic partnerships with anaerobic methanotrophs. This genomic evidence shows a possible mechanism for direct electron transfer from methanotrophic archaea into the metabolism of the partner bacteria.

## INTRODUCTION

The anaerobic oxidation of methane (AOM) is mediated by syntrophic consortia of anaerobic methanotrophic (ANME) archaea and deltaproteobacterial sulfate-reducing bacteria (SRB) and is the dominant mechanism for controlling the flux of methane from marine sediments (1–3). Since the initial molecular microbial ecology studies describing the association between methane-oxidizing ANME archaea and sulfate-reducing *Deltaproteobacteria* were published (2, 3), significant effort has been devoted to understanding the genetic potential of the ANME archaea and the mechanism enabling this unusual syntrophic partnership using isotopic (2, 3), metagenomic (4, 5), metatranscriptomic (5, 6), and metaproteomic (7, 8) analyses in natural samples and reactor systems. Initial hypotheses focused on conventional syntrophic substrates such as hydrogen, formate, and acetate as diffusible intermediates; however, experimental amendment of these compounds into sediment microcosms failed to decouple the syntrophic association (9–11). Recent experimental and molecular evidence supports an alternative hypothesis based on extracellular electron transfer (EET), using multiheme cytochromes to pass electrons produced during methane oxidation by the ANME archaeon directly to its bacterial partner (12, 13). The process of EET has been studied rigorously in metal-reducing organisms from the genera *Shewanella* and *Geobacter*, where electrons are transferred to extracellular metals (14) or syntrophic microorganisms (15, 16) using outer membrane multiheme cytochromes and/or conductive nanowires. Comparative genomics has shown that ANME-2 genomes contain very large multiheme cytochromes (13, 17) that are similar in size to the outer membrane cytochromes used by *Geobacter* and *Shewanella* to respire metals or to grow on electrode surfaces. Staining for heme and redox active proteins in ANME-2:deltaproteobacterium consortia showed localization in the extracellular matrix between cells (13), while electron microscopy of thermophilic ANME-1 consortia revealed extracellular structures produced by the “HotSeep-1” sulfate-reducing partner ("*Candidatus* Desulfofervidus auxilii") that visually resemble nanowires produced during EET by *Geobacter* (12). This observation was further supported by metatranscriptomic data showing upregulation of pili and outer membrane cytochromes by "*Ca*. Desulfofervidus auxilii" during syntrophic growth with ANME archaea (12). Finally, microcosm experiments revealed that high rates of methane oxidation by ANME-2 can occur without sulfate in the presence of alternative extracellular electron acceptors, such as 9,10-anthraquinone-2,6-disulfonate (AQDS), iron citrate, and humic acids substituting for an active SRB partner (18).

The majority of research to date has focused on the metabolism of ANME archaeal lineages; however, there have been fewer studies on the diversity of and metabolic potential within the associated SRB partners in methane seeps. The syntrophic partners of ANME archaea come from a number of environmental clades of *Deltaproteobacteria*. The most common partner bacteria, known as SEEP-SRB1 (19), are most closely related to *Desulfosarcina* and *Desulfococcus* (20); however, multiple other clades within *Desulfobulbaceae* have been shown to form associations with ANME archaea (19, 21–24). To date, genomic analysis of ANME partners has been restricted to "*Ca*. Desulfofervidus auxilii," a representative from the “HotSeep1” clade, which can form syntrophic associations with thermophilic members of ANME-1 and is distantly related to the common deltaproteobacterial partners of ANME archaea from globally distributed cold seep sediments (25, 26). "*Ca*. Desulfofervidus auxilii" is able to grow without the ANME-1 partner using hydrogen as the electron donor (26), which has not been demonstrated with SEEP-SRB1 partners. Recent sequencing of the "*Ca*. Desulfofervidus auxilii" genome confirmed the presence of periplasmic hydrogenases and of multiheme cytochromes that may be involved in extracellular electron transfer with ANME-1 archaea (26). The current lack of genomic data for the more widely distributed deltaproteobacterial partners of the ANME archaea in cold sediments makes it difficult to assess whether these traits are universal among the bacteria that form syntrophic partnerships with ANME archaea. Here we reconstructed genomes from diverse ANME partner bacteria belonging to the SEEP-SRB1 clade across multiple continental margin seep environments. Genomic comparisons between these ANME partners and cultured *Deltaproteobacteria* species revealed a number of unique genomic features in the syntrophic ANME partners that are suggestive of a common ability to engage in interspecies extracellular electron transfer.

## RESULTS AND DISCUSSION

**FIG 1:**
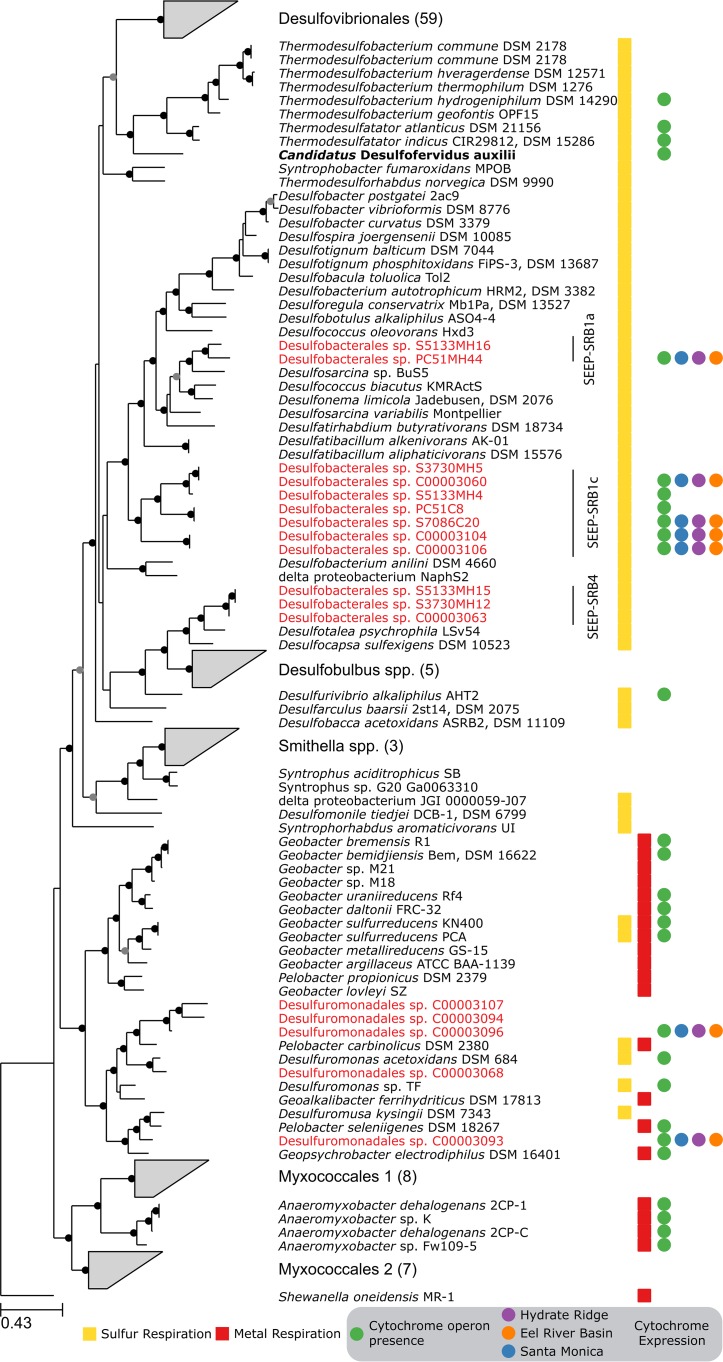
Phylogeny of methane seep *Deltaproteobacteria* and related organisms. Maximum likelihood phylogeny data were determined on the basis of an alignment of 40 universally conserved protein sequences. Internal nodes in the tree with greater than 70% or 90% bootstrap support are marked by gray or black circles, respectively. Genome bins identified in our metagenomic sequencing are highlighted in red; many of them are grouped into the *Desulfobacteraceae* SEEP-SRB1 or the *Desulfobulbaceae* SEEP-SRB4 clades. "*Ca*. Desulfofervidus auxilii," a previously analyzed ANME partner, is shown in bold text. Wedges represent multiple related genomes that have been collapsed for brevity. At the right, yellow squares indicate that the organism is capable of respiration using any sulfur compound; red squares indicate the ability to perform metal respiration; green circles indicate organisms that contain the same cytochrome-containing operon that is found in SEEP-SRB1. Blue, purple, and orange circles indicate that one or more of the four core genes in the cytochrome operon had been detected in the Santa Monica Mounds, Hydrate Ridge, or Eel River Basin sites.

Shotgun metagenomic sequencing of five sediment samples from Hydrate Ridge and Santa Monica Basin seep sites resulted in 8 draft genomes from diverse members of the most common, globally distributed *Desulfobacteraceae* clade, SEEP-SRB1, and 10 additional genomes from members of *Desulfuromondales*, *Desulfovibrio*, and *Desulfobulbaceae* (Fig. 1; see also Fig. S1 in the supplemental material). The levels of completeness and contamination, based on the presence of single-copy marker genes, ranged between 65% and 90% and between 1% and 15%, respectively (see Table S1 in the supplemental material). The SEEP-SRB1 group represented two previously described subclades, SEEP-SRB1a and SEEP-SRB1c (19), and had an average level of amino acid identity between genomes of 60%, indicating that each genome represented a unique family-level taxonomic classification (27) (see Text S1 in the supplemental material).

10.1128/mBio.00530-17.2 FIG S1  Maximum likelihood phylogenetic tree of the 16S rRNA gene from SEEP-SRB1. Gray wedges represent clades of sequences, whose numbers are shown inside the wedges. The three genome bins that contained 16S rRNA genes are labeled in red. Download FIG S1, PDF file, 0.2 MB. Copyright © 2017 Skennerton et al. 2017 Skennerton et al. This content is distributed under the terms of the Creative Commons Attribution 4.0 International license.

10.1128/mBio.00530-17.7 TABLE S1  Basic assembly statistics for genome bins reported in this study. Download TABLE S1, XLSX file, 0 MB. Copyright © 2017 Skennerton et al. 2017 Skennerton et al. This content is distributed under the terms of the Creative Commons Attribution 4.0 International license.

10.1128/mBio.00530-17.1 TEXT S1  Additional information from the genome bins recovered in this study and metabolic reconstruction of SEEP-SRB4 clade genomes. Download TEXT S1, DOCX file, 0.01 MB. Copyright © 2017 Skennerton et al. 2017 Skennerton et al. This content is distributed under the terms of the Creative Commons Attribution 4.0 International license.

**FIG 2:**
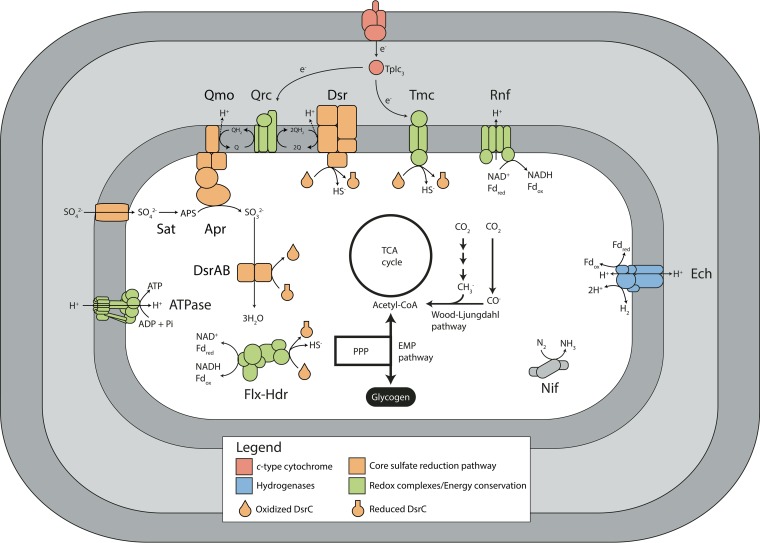
Proposed model of SEEP-SRB1 metabolism in AOM consortia. All identified SRB from AOM consortia contained the canonical sulfate reduction pathway: sulfate adenylyltransferase (Sat), adenylyl-sulfate reductase (Apr), dissimilatory sulfite reductase (DsrAB), and the membrane-associated complexes Qmo and DsrMKJOP. The DsrC protein acts as a key intermediate for transferring electrons from DsrAB to other redox-active complexes, including the DsrMKJOP and Tmc membrane complexes and the soluble Flx-Hdr complex. Electrons required to reduce the Qrc or Tmc membrane complexes are proposed to be sourced from direct extracellular electron transfer from the ANME archaeon cell mediated by outer membrane *c*-type cytochromes. All genomes fix carbon using the Wood-Ljungdahl pathway and contain a complete tricarboxylic acid (TCA) cycle, a pentose phosphate pathway (PPP), and the Embden-Meyerhof-Parnas (EMP) pathway for glycolysis/gluconeogenesis.

Despite the broad phylogenetic diversity, all SEEP-SRB1 genomes contained hallmarks of an autotrophic lifestyle, including carbon and nitrogen fixation (Fig. 2). The genomes contained the Wood-Ljungdahl pathway (a reductive acetyl-coenzyme A [CoA] pathway) for carbon fixation in agreement with previous observations from lipid biomarkers and [^13^C]bicarbonate labeling studies (28); in contrast, "*Ca*. Desulfofervidus auxilii" utilizes the reductive TCA cycle (26). The genomes contained the Embden-Meyerhof-Parnas pathway and the pentose phosphate pathway, which links carbon fixation to biomass and carbohydrate synthesis and enables the generation of glycogen as a storage compound. Previous ecophysiological studies of AOM consortia in methane seep sediments have demonstrated differences in nitrogen utilization among different ANME partners, including direct or indirect involvement in nitrogen fixation (29, 30) and nitrate utilization (23). The genomic data support these findings, with nitrogenase identified in the SEEP-SRB1 genome bins, suggesting that N_2_ can be used as a biosynthetic nitrogen source.

The SEEP-SRB1 genomes contain all of the genes necessary for the canonical sulfate reduction pathway, including those encoding sulfate adenylyl transferase (Sat), adenosine phosphosulfate (APS) reductase (AprAB), dissimilatory sulfite reductase (DsrAB), and sulfur carrier protein DsrC (31) (Fig. 2). All of the genome bins contained genes for the QmoABC and DsrJKMOP membrane complexes, which are required for sulfate reduction. QmoABC donates electrons from quinone to APS reductase (32), whereas DsrMKJOP donates electrons from the quinone pool to produce sulfide by breaking the trisulfide intermediate of DsrC formed by the action of the dissimilatory sulfite reductase (31, 33). In addition to genes for the core sulfate reduction pathway, the SEEP-SRB1 genome bins contained genes for the Tmc transmembrane spanning complex (34) and the cytoplasmic Flx-Hdr complex (35) (Fig. 2), which are widely distributed in deltaproteobacterial SRB (36) and may contribute to redox balance by interacting with DsrC. The Tmc complex has been shown to accept electrons from soluble periplasmic cytochrome *c* and is hypothesized to transfer electrons to DsrC to generate sulfide (34). Energy conservation involving the Flx-Hdr complex is proposed to occur through flavin-based electron bifurcation by oxidizing NADH that is coupled to the unfavorable reduction of ferredoxin to the (hypothesized) favorable reduction of DsrC (35). In *Desulfovibrio vulgaris* Hildenborough, Flx-Hdr is essential for NADH oxidation during growth on ethanol (35).

Quinones are a vital part of the SRB respiratory chain, donating electrons to APS reductase in the second step of sulfate reduction and to the DsrJKMOP membrane complex in the final step of sulfate reduction. The ability to synthesize respiratory quinones was found in the SEEP-SRB1 genomes, which also carry the genes that encode the quinone reductase complex (QrcABCD) (37), consistent with most other members of the *Desulfobacteraceae*. This membrane-bound complex is believed to play a role in energy conservation by transferring electrons from soluble periplasmic cytochrome *c* into the quinone pool.

In model sulfate reducers, such as *Desulfovibrio vulgaris*, reducing power for the Qrc and Tmc membrane complexes is sourced from periplasmic hydrogenases or formate dehydrogenases using a small soluble cytochrome *c* (TpIc_3_) as a periplasmic electron shuttle (38). Notably, all of the SEEP-SRB1 genomes appear to lack both periplasmic hydrogenases and formate dehydrogenases but do contain the membrane-bound energy-converting hydrogenase (Ech) and cytoplasmic formate dehydrogenases. There were a number of operons that included genes similar to those encoding electron-transferring subunits of the Hox-type or F420-reducing multisubunit hydrogenases; however, none of these operons contained genes encoding the hydrogenase subunit. Ech has been biochemically characterized in *Methanosarcina barkeri*, where it can generate reduced ferredoxin (while consuming H_2_) during methanogenesis using H_2_/CO_2_ or oxidize ferredoxin (while producing H_2_) during acetoclastic methanogenesis (39). The physiological role of Ech in sulfate reducers is currently unknown and may be species specific; for example, Ech plays a minor role in overall bioenergetics in *Desulfovibrio gigas* (40), whereas Ech is highly upregulated using H_2_ as the electron donor in *Desulfovibrio vulgaris* (41). The lack of periplasmic hydrogenases or formate dehydrogenases helps explain why efforts to grow SEEP-SRB1 organisms using H_2_ or formate have failed (9, 10). In contrast, "*Ca*. Desulfofervidus auxilii" contains a periplasmic hydrogenase and is capable of growing using H_2_ in the absence of an ANME partner (12, 26).

**FIG 3:**
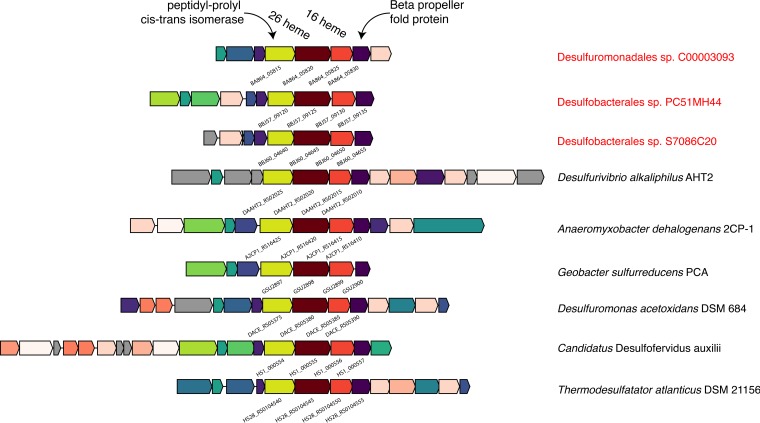
Representative operon structure from organisms containing large multiheme cytochromes found in SEEP-SRB1. Homologous genes are colored the same between organisms, with the exception of the cytochromes, which are colored with various intensities of red based on the number of heme binding motifs present in the gene. Genes in gray are not conserved (i.e., are unique to that genome). The NCBI locus tag identifier for the core set of four genes is shown below each operon.

Without periplasmic hydrogenases or formate dehydrogenases, SEEP-SRB1 organisms must have alternative mechanisms to reduce membrane complexes used in respiration. Recent evidence has pointed to electrons being transferred from ANME archaea to SRB partners using large extracellular multiheme cytochromes (12, 13, 17). The SRB partners must have a complementary mechanism that enables these electrons to participate in their metabolism. Analyses of syntrophic *Geobacter* species, and of a partnership of *Geobacter* and *Methanosaeta*, performing extracellular electron transfer have identified type IV pili and multiheme cytochromes as crucial to the transfer of electrons between partners (15, 42). Similarly, the SEEP-SRB1 genomes included type IV pili and unusually large multiheme cytochromes (Fig. 3; Fig. S2), many of which contained homologs to *Geobacter* but not to other sulfate reducers (Fig. S3). The largest cytochrome in most previously characterized sulfate-reducing *Deltaproteobacteria* species is encoded by the *hmcA* gene and contains 16 heme binding motifs. In comparison, the *Desulfobacteraceae* SEEP-SRB1a and SEEP-SRB1c genome bins contained an operon with two multiheme cytochromes, including one with 26 heme binding motifs that was adjacent to another with 16 heme binding motifs that is unrelated to *hmcA* (Fig. 3). There were many additional families of cytochromes that were present in the SEEP-SRB1 with homologs in the *Desulfuromonadales* but not in previously sequenced free-living SRB (Fig. S3). A second operon, also widely distributed in SEEP-SRB1, included two cytochromes with 11 or 12 heme binding motifs that were related to the OmcX gene in *Geobacter*. The genome bins from SEEP-SRB4, though not known to be ANME partners, also contained an operon with a multiheme cytochrome containing 19 heme binding motifs that was distinct from those seen with SEEP-SRB1 and all other cultured *Deltaproteobacteria* species (see Text S1 in the supplemental material).

10.1128/mBio.00530-17.3 FIG S2  Cytochrome *c* numbers and sizes in *Deltaproteobacteria* and *Thermodesulfobacterales*. The total number of examples of cytochrome *c*, judged as protein sequences containing a heme binding domain, is shown as a fraction of the total number of genes in the genome along the *x* axis. The size of the largest cytochrome in each genome is shown on the y axis. ANME partner bacteria are highlighted as large stars. Download FIG S2, PDF file, 0.3 MB. Copyright © 2017 Skennerton et al. 2017 Skennerton et al. This content is distributed under the terms of the Creative Commons Attribution 4.0 International license.

10.1128/mBio.00530-17.4 FIG S3  Comparison of multiheme cytochromes in *Desulfobacterales*, *Desulfomonadales*, *Desulfovibrionales*, and *Thermodesulfobacterales*. Homologous cytochromes with more than 4 heme binding motifs are shown with the ortholog identifier given by proteinortho. The count represents the number of instances of each particular ortholog in each genome (a count value greater than 1 suggests the presence of paralogs). Genomes highlighted in red are known to show syntrophy with ANME archaea. Download FIG S3, PDF file, 0.3 MB. Copyright © 2017 Skennerton et al. 2017 Skennerton et al. This content is distributed under the terms of the Creative Commons Attribution 4.0 International license.

Homologs of the SEEP-SRB1 cytochromes are present only in some other cultured *Deltaproteobacteria* species, predominantly in the *Desulfuromonadales* that are known metal reducers, including genome bins from the sediment samples (Fig. 1). Additionally, "*Ca*. Desulfofervidus auxilii" (26), several related *Thermodesulfobacterales* species, *Anaeromyxobacter*, and *Desulfurivibrio alkaliphilus* AHT2 (43) contain homologs of these cytochromes. This operon contained a core set of four genes encoding a six-bladed beta propeller fold protein, the two cytochromes (16 heme and 26 heme), and a peptidyl-prolyl *cis*-*trans*-isomerase protein. These four proteins contained signal peptides localizing them to the periplasm but did not contain any transmembrane helices, suggesting that they were not physically attached to either the inner or outer membrane. Surrounding these core genes were open reading frames encoding proteins that vary in composition and number but are often smaller cytochromes, proteins containing beta propeller fold motifs, and proteins of unknown function, some of which contained transmembrane helices (Fig. 3). *D. alkaliphilus* AHT2 contains two copies of this operon that do not appear to be the result of a recent internal duplication event as each operon contains a different complement of additional cytochromes that differ in the number of heme binding motifs.

Electron transfer across the outer membrane is achieved in a number of organisms by using a porin-cytochrome complex (44–46). These complexes consist of a porin-like integral outermembrane protein and at least one cytochrome which uses the pore to traverse the membrane. The SEEP-SRB1a and SEEP-SRB1c genomes did not contain any homologs of previously identified porins specifically from known porin-cytochrome complexes. However, the genomes did contain open reading frames annotated as encoding homologs of OmpA/OmpF outer membrane porins. It is possible that SEEP-SRB1 genomes utilize a novel mechanism to transfer electrons across the outer membrane or that they encode a porin that is not related to those previously identified in other organisms performing extracellular electron transfer.

**FIG 4:**
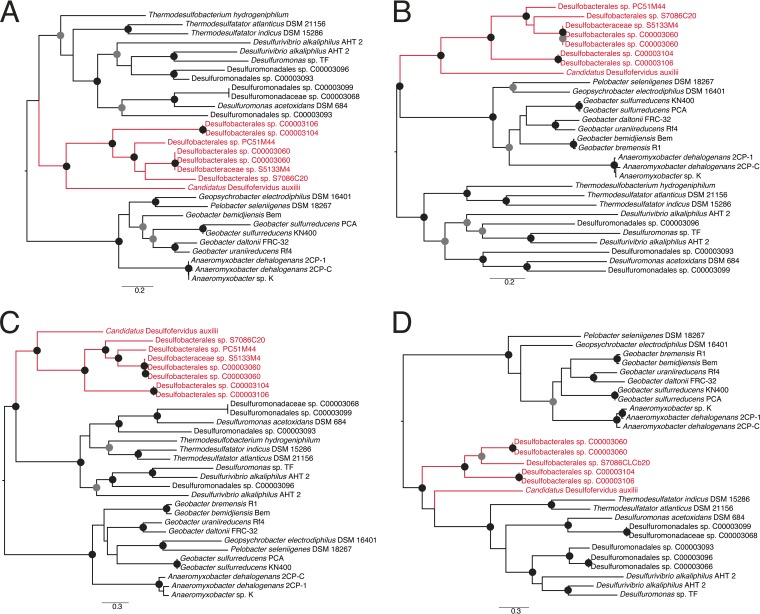
Maximum likelihood phylogenetic trees of (A) the 16-heme cytochrome; (B) the 26-heme cytochrome; (C) the peptidyl-prolyl *cis*-*trans*-isomerase; and (D) the six-bladed beta propeller fold protein. Each tree was rooted at the midpoint branch. Internal nodes in the tree with greater than 70% or 90% bootstrap support are marked by gray or black circles, respectively. The ANME partners are labeled in red. Scale bars represent numbers of substitutions per site.

Phylogenies of the conserved four proteins showed that the genomes belonging to SEEP-SRB1 and the distantly related "*Ca*. Desulfofervidus auxilii" grouped together in what appears to be an ANME partner-associated clade (Fig. 4). Two other clades were present; the first was composed mostly of *Geobacter* and *Anaeromyxobacter*, and the second contained *Desulfuromonas* and related organisms that included genome bins from the seep sediments, *Desulfurivibrio alkaliphilus* AHT2, and the other members of the *Thermodesulfobacteriales*. The phylogenies suggest that "*Ca*. Desulfofervidus auxilii" may have obtained its operon from the common ancestor of the SEEP-SRB1 clade, whereas the other *Thermodesulfobacterales* appear to have obtained their cytochromes from members of the *Desulfuromonadales*.

There is no known physiological function for the homologs of these cytochromes in cultured organisms, and so the significance of these clades cannot be determined. Proteomic and transcriptomic experiments in *Anaeromyxobacter dehalogenans* 2CP-C (47), *Geobacter sulfurreducens* (48), *Geobacter bemidjiensis* (49), and *Desulfuromonas acetoxidans* (50) showed that these cytochromes are not expressed during metal respiration. However, metal respiration donates electrons to the extracellular environment rather than accepting them from external sources. A number of microbes can accept electrons from electrodes, including *G. sulfurreducens* (42, 51–54). In a microarray study performed using *G. sulfurrenducens* growing on the cathode, the homologs to the cytochrome-encoding operon detected in SEEP-SRB1 did not show significantly different expression results under current-producing or -consuming conditions (55). However, the experimental conditions of this study poised the electrode at −500 mV, which is significantly more negative than sulfate (−220 mV), the terminal electron acceptor for SEEP-SRB1. We hypothesize that this operon is expressed in situations with an extracellular electron donor that is similar in redox potential to that supplied by the ANME archaea, which must have a more positive redox potential than sulfate.

The expression of this operon was assessed using semiquantitative metaproteomics *in situ* at methane seeps at the Santa Monica Mounds, Eel River Basin, and Hydrate Ridge along the west coast of North America (Fig. S4; Table S2). While absolute quantification of expressed proteins was beyond the scope of current study, a semiquantitative mass spectrometry (MS) approach was taken to assess the relative abundances of expressed proteins and has been used in a number of previous protein expression analyses of complex samples (56–58). The raw mass spectra were matched against a large concatenated predicted proteomic database containing 16 genomes that belonged to *Desulfobacteraceae* SEEP-SRB1, *Desulfobulbaceae* SEEP-SRB4, and *Desulfuromondales* genomes recovered from the seep sediments. Although the total number of identified proteins was modest (367 proteins across all three sites), among these were informative proteins that belonged to the Wood Ljungdahl pathway (carbon monoxide dehydrogenase, formylmethanofuran-tetrahydromethanopterin *N*-formyltransferase) and to nitrogen fixation and sulfate reduction pathways (Sat, AprAB, DsrAB, and DsrC) (Table S2). The identification of multiheme cytochrome proteins, which are abundant at low to moderate levels in these systems, was challenging. For complex systems, metaproteomics easily identifies the most abundant proteins but is limited in dynamic range; thus, the lower-abundance proteins often do not have adequate fragment ions or signal intensity to pass the standard threshold filters (59, 60). In particular, the use of a large database to search for low-abundance proteins adds to the difficulty in identifying the less abundant proteins, since overall peptide identification metrics (spectral matching, scoring criteria, and false-discovery rates [FDR]) are driven by the higher-abundance peptides/proteins (61). To better enable investigations using the search criteria for scouting out lower-abundance proteins, the database complexity was reduced by generating a smaller database of 2,246 proteins derived from the 16 genomes of SEEP-SRB1 and SEEP-SRB4 clades. This database comprised proteins with four or more heme motifs and the full genome of *Desulfuromonadales bacterium* C00003107 (consisting of genes encoding ~2,000 proteins). Using this approach, we could detect several multiheme cytochromes, all having fewer than 10 heme motifs (Table S3). For a more refined search, we further reduced the database complexity by assembling another database comprising multiheme cytochromes only, derived from the 16 genomes of SEEP-SRB1 and SEEP-SRB4 clades. Protein expression was detected in all three sites from members of SEEP-SRB1a and SEEP-SRB1c and two members of the *Desulfomonadales* that were also recovered from the seep sediment metagenomes (Fig. 1; Table S4). Not all genes from each operon were detected at all sites, with Santa Monica and Eel River Basin having more matches than Hydrate Ridge (Table S4). To more definitively support these limited database searches, peptide and protein identification was confirmed by manual validation of acquired peptide mass spectra from representatives of all the four members that constitute this operon (Fig. S5). These results show that these cytochromes are expressed *in situ* in syntrophic partnership with ANME archaea but at levels that are lower than those of enzymes from the sulfate reduction pathway. The reasons for this are unclear; it may be that extraction and detection were more difficult or that cells may not require that many copies of the protein or may be involved in a separate physiology not related to ANME syntrophy. However, their presence in SEEP-SRB1 and the phylogenetically unrelated "*Ca*. Desulfofervidus auxilii" as a possible component of the ANME archaeon-SRB syntrophy warrants further analysis.

10.1128/mBio.00530-17.5 FIG S4  Map of the west coast of United States showing the sample sites for metaproteomic sampling. Download FIG S4, PDF file, 0.04 MB. Copyright © 2017 Skennerton et al. 2017 Skennerton et al. This content is distributed under the terms of the Creative Commons Attribution 4.0 International license.

10.1128/mBio.00530-17.6 FIG S5  Spectra for manually identified peptides from possible extracellular electron transfer operons. The b-type and y-type ions of the peptide are shown in purple and blue, respectively. The amino acid sequence of the peptide is shown in the upper left corner. Download FIG S5, PPT file, 0.3 MB. Copyright © 2017 Skennerton et al. 2017 Skennerton et al. This content is distributed under the terms of the Creative Commons Attribution 4.0 International license.

10.1128/mBio.00530-17.8 TABLE S2  Normalized spectral counts for proteins identified from a database of 16 genomes identified in this study. Download TABLE S2, XLSX file, 0.04 MB. Copyright © 2017 Skennerton et al. 2017 Skennerton et al. This content is distributed under the terms of the Creative Commons Attribution 4.0 International license.

10.1128/mBio.00530-17.9 TABLE S3  Normalized spectral counts for multiheme cytochromes detected using a medium-size database containing proteins with four or more heme binding motifs. Download TABLE S3, XLSX file, 0.03 MB. Copyright © 2017 Skennerton et al. 2017 Skennerton et al. This content is distributed under the terms of the Creative Commons Attribution 4.0 International license.

10.1128/mBio.00530-17.10 TABLE S4  Normalized spectral counts for the four core genes from the possible extracellular electron transfer operons. Download TABLE S4, XLSX file, 0.04 MB. Copyright © 2017 Skennerton et al. 2017 Skennerton et al. This content is distributed under the terms of the Creative Commons Attribution 4.0 International license.

In model SRB, a small soluble periplasmic cytochrome *c* (TpIc_3_) is central to transferring electrons from periplasmic hydrogenases or formate dehydrogenases to membrane complexes (36). It is therefore likely that the transfer of ANME archaeon-derived electrons to TpIc_3_ occurs via outer membrane cytochromes as this minimizes the overall changes in the metabolic network. This same scheme has been suggested in “*Ca*. Desulfofervidus auxilii” as it could allow easy switching between syntrophic growth and hydrogenotrophic growth (26). The metabolic flexibility of “*Ca*. Desulfofervidus auxilii” may suggest an evolutionary route that SEEP-SRB1 organisms might have undertaken to become syntrophic partners of ANME archaea. The first stage may have been the acquisition of large multiheme cytochromes that allowed extracellular electrons to enter the cell. At this stage, the SRB could easily transition between independent hydrogenotrophic growth and syntrophy with amenable partners, much as “*Ca*. Desulfofervidus auxilii” is capable of doing. The syntrophy may have become obligate in SEEP-SRB1 through the loss of genes encoding periplasmic hydrogenases and formate dehydrogenases that could act as alternative electron donors. This gene loss may explain why members of SEEP-SRB1 have not been cultured or enriched from hydrogen- or formate-amended microcosm experiments. One future avenue for culture of SEEP-SRB1 may be that of using electrodes or other extracellular electron donors to replace the role of ANME archaea in donating electrons to the SRB.

## MATERIALS AND METHODS

### Metagenome sample collection.

Five sediment samples from Hydrate Ridge or the Santa Monica Mounds off the Pacific coast of United States were used for metagenome sequencing. Sample collection details for three samples from Hydrate Ridge, labeled 3730, 5133-5, and 5579, have been described previously (62). Briefly, sample 3730 was collected within a Calyptogena clam bed from Hydrate Ridge south (44°43.09′N, 125°9.14′W; depth, 776 m); samples 5133-5 and 5579 were collected from a white microbial mat at Hydrate Ridge North, station 7 (44°40.03′N, 125°6.00′W; depth, 600 m). Two additional samples were collected from the Santa Monica Basin, offshore in California, as part of the *R/V Western Flyer* Southern California Expedition in May 2013. Using the *ROV Doc Ricketts*, a sediment push core, PC51 (33°47.3301′N, 118°40.0979′W), was collected on dive DR-461 at a depth of 863 m from the Santa Monica Mounds, characterized by the presence of a white microbial mat. Upon recovery shipboard, PC51 was separated into two samples that were used for metagenomic sequencing. Sample 7086 was created from sediment in the 6-cm to 9-cm horizon of the push core. The remaining sediment samples (0 to 6 cm; 9 to 15 cm) were combined and used as the sample PC51 mix. Aliquots of each were preserved for DNA extractions at −80°C prior to sequencing. DNA was extracted from the sediment using a Powersoil DNA extraction kit (catalog no. 12888; Mo Bio Laboratories, Inc., Carlsbad, CA).

### Metaproteome sample collection, processing, and analysis.

Five sediment samples were collected for metaproteomics from three locations. A 20-cm push core (PC48) was collected on 26 July 2005 from the Eel River Basin on dive T-863 of *R/V Western Flyer* using *ROV Tiburon* (coordinates 40°48.6631′N, 124°36.7437′W; water depth, 520 m) and divided into two sections of 10 cm (0 to 10 cm and 10 to 20 cm). A second 20-cm push core was collected on 15 February 2005 on dive T-796 of *R/V Western Flyer* using *ROV Tiburon* from a mound a few hundred meters northwest of the venting mound in Santa Monica Basin (coordinates 33°47.9748′N, 118°38.796′W; water depth, 826 m). This push core was divided into 4-cm segments; the 0-cm to 4-cm horizon and 8-cm to 12-cm horizon were used for proteomics. The final sample was the sediment 3730 sample collected from Hydrate Ridge that was also used for metagenomic sequencing.

### Cellular lysis, protein extraction, and sample preparation.

Partially thawed seep sediments (5 g) were suspended in 10 ml of detergent-based lysis buffer and subjected to heat-assisted cellular lysis as described previously (63). The suspension was cooled on the benchtop and centrifuged in fresh tubes for 5 min at 8,000 × *g* to settle the sediment. The resulting clear supernatant was aliquoted into fresh tubes and amended with chilled 100% trichloroacetic acid (TCA) to a final concentration of 25% (vol/vol) and kept at −20°C overnight. The residual sediment was discarded. Following overnight TCA precipitation, the supernatant was centrifuged at 21,000 × *g* for 20 min to obtain a protein pellet. The pellet was retained, washed thrice with chilled acetone (64), air dried, and solubilized in 6 M guanidine buffer (6 M guanidine, 10 mM dithiothreitol [DTT], Tris-CaCl_2_ buffer [50 mM Tris; 10 mM CaCl_2_; pH 7.8]) and incubated at 60°C for 3 h with intermittent vortex mixing. An aliquot of 25 µl was utilized for protein estimation, which was carried out using an RC/DC protein estimation kit (Bio-Rad Laboratories, Hercules, CA) per the manufacturer’s instructions. The remaining protein sample was diluted 6-fold using Tris-CaCl_2_ buffer, and trypsin was added (40 µg/1 to 3 mg total protein) based on protein estimation results. Proteins were digested overnight at 37°C with gentle mixing, and the resulting peptides were reduced by addition of DTT (10 mM) and desalted using a Sep-Pak column and solvent exchange (65). Peptides were stored at 80°C until MS analysis was performed.

All chemicals used in sample preparation and mass spectrometry analysis were obtained from Sigma Chemical Co. (St. Louis, MO), unless mentioned otherwise. Sequencing-grade trypsin was acquired from Promega (Madison, WI). High-performance liquid chromatography (HPLC)-grade water and other solvents were obtained from Burdick & Jackson (Muskegon, MI), and 99% formic acid was purchased from EM Science (Darmstadt, Germany).

### NanoLC-MS/MS analysis.

Peptide mix (100 µg peptide) was pressure loaded onto a biphasic resin-packed column (SCX [Luna; Phenomenex, Torrance, CA] and C_18_ [Aqua; Phenomenex, Torrance, CA]) as described earlier (65, 66). The sample column was connected to the C_18_ packed nanospray tip (New Objective, Woburn, MA) mounted on a Proxeon (Odense, Denmark) nanospray source as described earlier (67). Peptides were chromatographically sorted using an Ultimate 3000 HPLC system (Dionex, USA) over the course of 24 h. The HPLC system was connected to a LTQ Velos mass spectrometer (Thermo Fisher Scientific, Germany), which was employed for peptide fragmentation and measurements via the Multi-Dimensional Protein Identification Technology (MuDPIT) approach as described earlier (65–67). The peptide fragmentation and measurements were carried out in data-dependent mode, using Thermo Xcalibur software V2.1.0. Each full scan (1 microscan) was followed by collision-activated dissociation (CID)-based fragmentation using 35% collision energy and the 10 most abundant parent ions (2 microscans) with a mass exclusion width of 0.2 m/z and a dynamic exclusion duration of 60 s.

### Bioinformatics and data analysis.

For protein identifications, the raw spectra were searched against three databases of various sizes via Myrimatch v2.1 (68) using parameters described previously (69). The first database was composed of 16 genomes identified in this study belonging to members of *Desulfobacterales* and *Desulfuromonadales*; the second database contained all predicted *c-*type cytochromes from these genomes containing four or more heme binding motifs (CxxCH amino acid sequences) and the open reading frames of *Desulfuromonadales* sp. strain C00003107; finally, the third database contained the core four proteins encoded by the cytochrome operon from all of the SEEP-SRB1 and *Desulfuromonadales* genomes recovered in this study. Static cysteine and dynamic oxidation modifications were not included in the search parameters. Identification of at least two peptides per protein sequence (one unique and one nonunique) was set as a prerequisite for protein identification. Common contaminant peptide sequences from trypsin and keratin were concatenated to the database along with reverse database sequences. The reverse database sequences were used as decoy sequences to calculate the false-discovery rate (FDR), which was maintained at <1% for the peptide-to-spectrum identification. For downstream data analysis, spectral counts of identified peptides were normalized as described before (70) to obtain the normalized spectral abundance factor (NSAF), also referred to as the normalized spectral count (nSpc). Averages of nSpc values from duplicate runs were used to obtain values of relative abundances of expressed proteins across different samples. Normalization of spectra helps account for differences in protein length and for variations in results of MS analysis of samples, thereby providing information on the relative abundances of proteins in a given sample and across samples in a given study.

### Metagenome sequencing and genome binning.

Sequencing, assembly, and binning of samples 3730, 5133-5, and 5579 have been described previously (62). Briefly, these samples were sequenced using a HiSeq 2000 system (Illumina, Inc., San Diego, CA) and assembled using CLC genomics workbench 6.0; metagenomic bins were defined using GroopM 0.2 (71). Additionally, a second assembly procedure was performed on the samples using megahit 0.3.3a (72), and the results were then binned using metabat 0.26.1 (73). This assembly and binning complemented the previous approach and resulted in additional genome bins. Sample 7086 and the PC51 mix sample were sequenced using an Illumina HiSeq 2500 system at the University of California, Davis. Raw metagenomic reads were assembled using both CLC genomics workbench 9.0 and megahit 1.0.3. Genome binning was performed on both of these assemblies using metabat 0.26.1.

### Comparative genomics.

Homologous proteins were identified across *Deltaproteobacteria* species with proteinortho 5.11 (74) using blast+ 2.2.30 (75). Orthologous groups relating to carbon fixation and energy metabolism were manually checked for inclusion of biochemically analyzed proteins and manually aligned using MUSCLE 3.8.31 (76) for inclusion of conserved amino acids, and the data were compared to the KEGG database using KAAS (77), the Uniprot database (78) and the Interpro database (79) to make sure that the correct annotations and protein motifs were present.

### Porin-cytochrome complex annotation.

Representative porin genes from *Geobacter sulfurreducens* (GSU2733), *Desulfurivibrio alkaliphilus* (DaAHT2_2270), and *Gallionella capsiferriformans* (Galf_2003) were used as search sequences against the SEEP-SRB1 genomes. BLASTP 2.2.29+ (75) was used with an E value cutoff of 0.01 to determine if there were any known homologs for these genes in SEEP-SRB1 genomes.

### Genome tree construction.

The genome tree was constructed from a concatenated alignment of 40 protein-coding genes that are universally distributed and in single-copy form in both archaea and bacteria (80). The marker genes were aligned to the hidden Markov model generated from each of the 40 marker genes using hmmer 3.1b2 (http://hmmer.org). A maximum likelihood tree was created using RAxML 8.1.7 (81) with the following settings: -f a -k -x 67842 -p 19881103 -N 100 -T 16 -m PROTGAMMAWAG. The tree was visualized using the ete toolkit (82).

### 16S rRNA gene tree construction.

A tree of the 16S rRNA gene was constructed of sequences belonging to SEEP-SRB1. A maximum likelihood tree was created using RAxML 8.1.7 (81) with the following settings: -f a -k -x 67842 -p 19881103 -N 100 -T 16 -m GTRGAMMAI.

### Data availability.

Raw sequencing data, metagenomic assemblies, and draft genome sequences are available under NCBI bioproject identifiers PRJNA326769 and PRJNA290197.
