# Erratum for Skennerton et al., “Methane-Fueled Syntrophy through Extracellular Electron Transfer: Uncovering the Genomic Traits Conserved within Diverse Bacterial Partners of Anaerobic Methanotrophic Archaea”

**DOI:** 10.1128/mBio.01561-17

**Published:** 2017-10-03

**Authors:** Connor T. Skennerton, Karuna Chourey, Ramsunder Iyer, Robert L. Hettich, Gene W. Tyson, Victoria J. Orphan

**Affiliations:** a Division of Geological and Planetary Sciences, California Institute of Technology, Pasadena, California, USA; b Chemical Sciences Division, Oak Ridge National Laboratory, Oak Ridge, Tennessee, USA; c Genome Science and Technology, University of Tennessee, Knoxville, Tennessee, USA; d Australian Centre for Ecogenomics, School of Chemistry and Molecular Biosciences, University of Queensland, Brisbane, Queensland, Australia

## ERRATUM

Volume 8, no. 4, e00530-17, 2017, https://doi.org/10.1128/mBio.00530-17. It has been brought to our attention that some of the gene labels assigned to *Geobacter sulfurreducens* are incorrect in Fig. 3. Upon careful review, we have confirmed that the analyses in our manuscript were performed on the correct genes, and it is only the figure labels that are incorrect. The revised Fig. 3 shows the correct labels.

**FIG 3:**
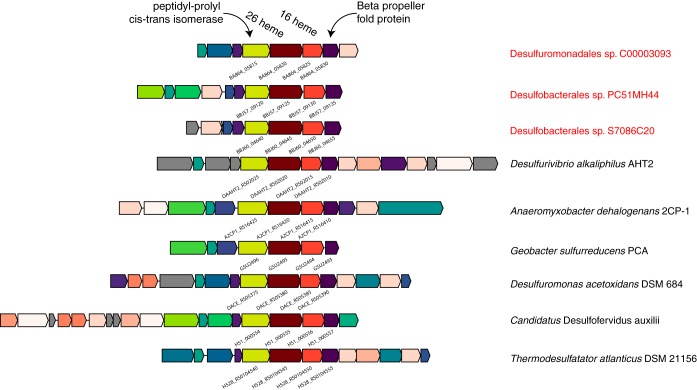
Representative operon structure from organisms containing large multiheme cytochromes found in SEEP-SRB1. Homologous genes are colored the same between organisms, with the exception of the cytochromes, which are colored in various intensities of red based on the number of heme-binding motifs present in the gene. Genes in grey are not conserved (unique to that genome). The NCBI locus tags for the core set of four genes are shown below each operon.

