# Supplementary figures and images for: Methane-Fueled Syntrophy through Extracellular Electron Transfer: Uncovering the Genomic Traits Conserved within Diverse Bacterial Partners of Anaerobic Methanotrophic Archaea
Source: mBio. 2017 Aug 1;8(4):e00530-17. doi: 10.1128/mBio.00530-17 (PMC5539420; doi:10.1128/mBio.00530-17)

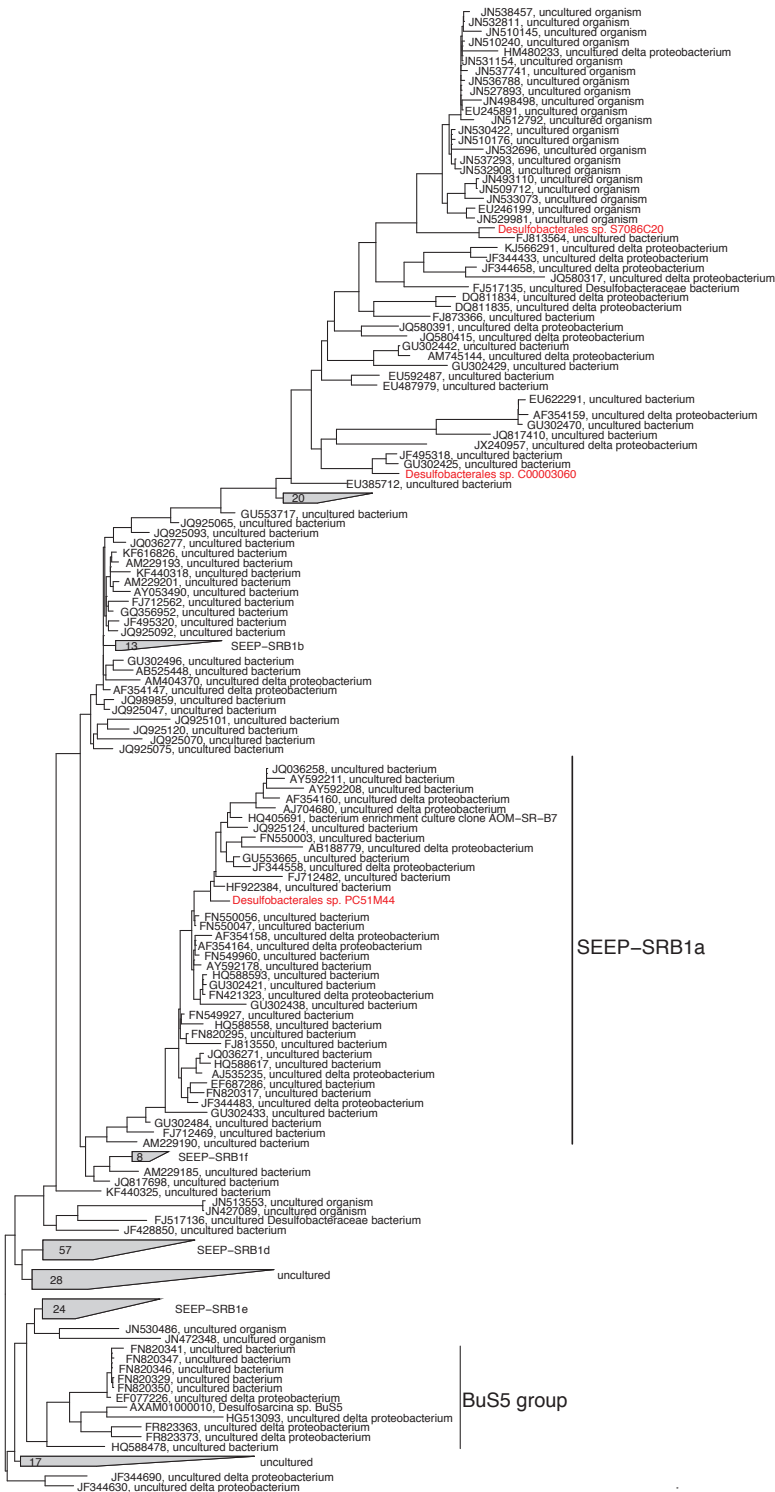

Supplement: FIG S1 [file mbo004173410sf1.pdf]

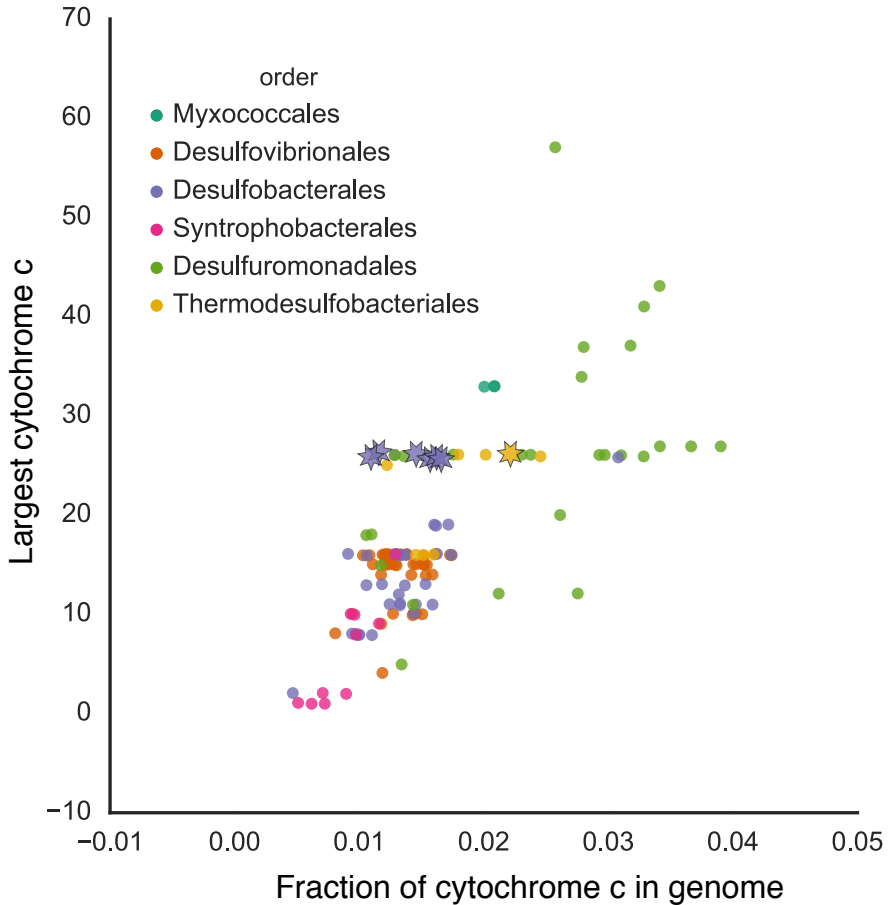

Supplement: FIG S2 [file mbo004173410sf2.pdf]

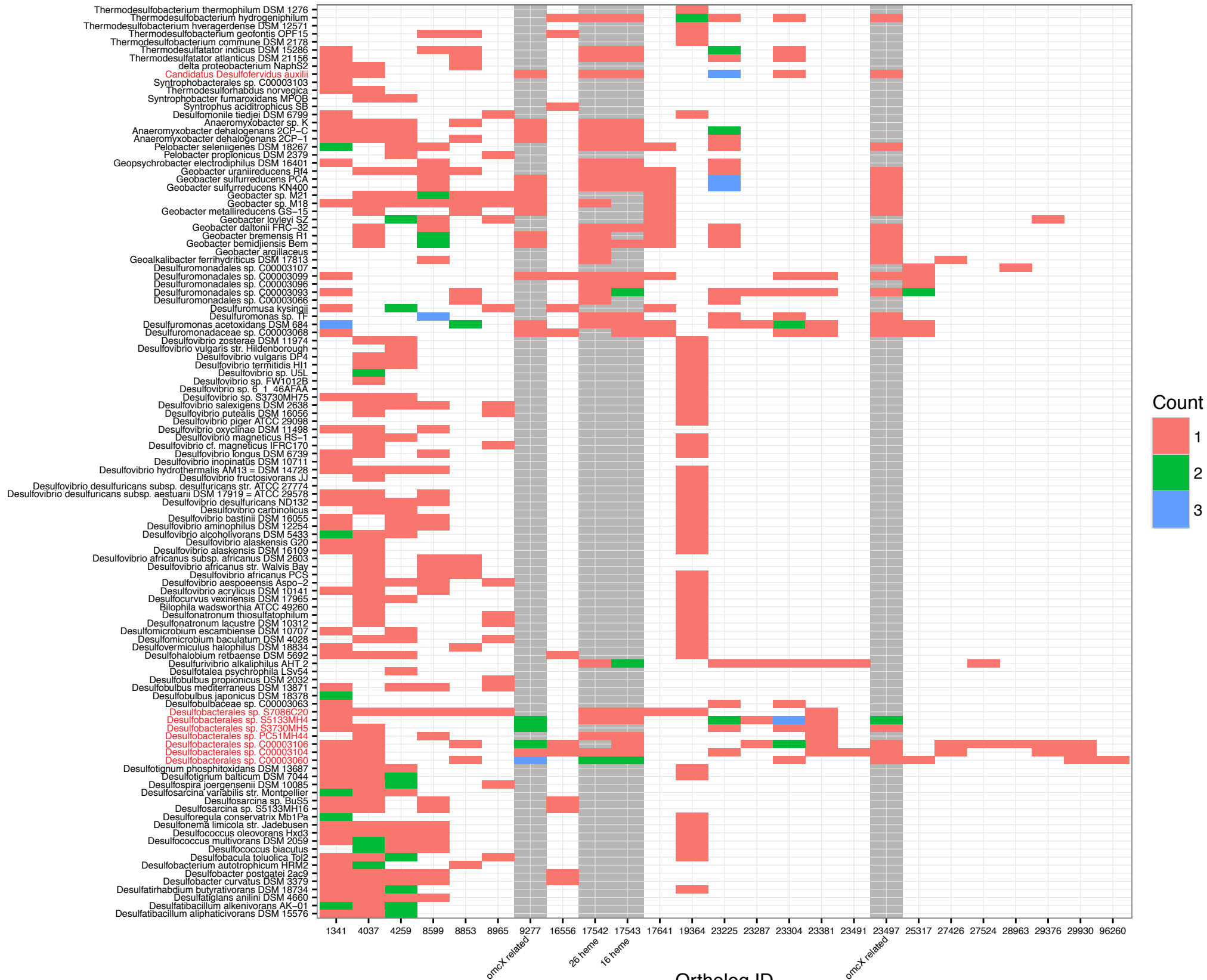

Supplement: FIG S3 [file mbo004173410sf3.pdf]

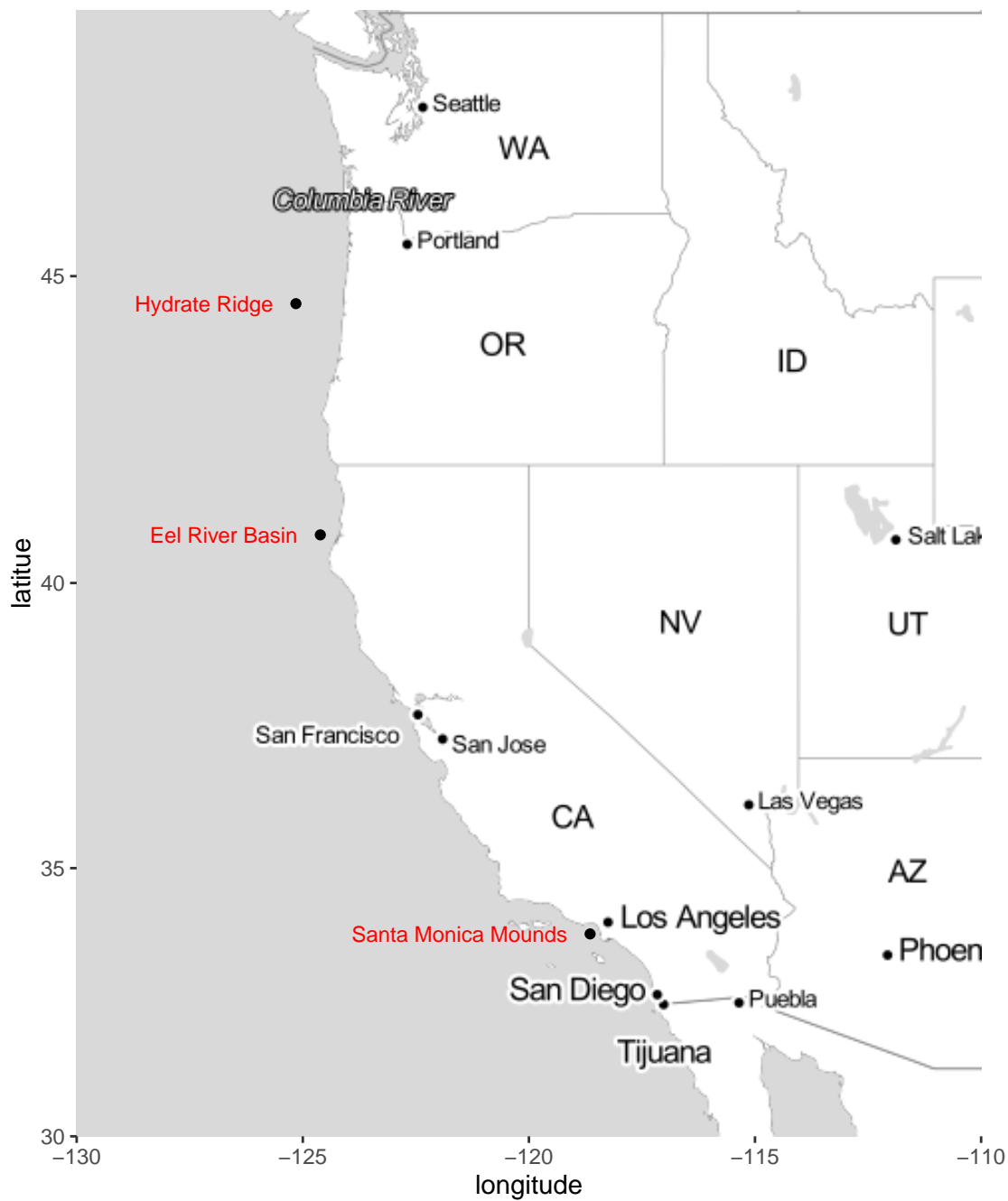

Supplement: FIG S4 [file mbo004173410sf4.pdf]
