# Supplementary material for: Methane-Fueled Syntrophy through Extracellular Electron Transfer: Uncovering the Genomic Traits Conserved within Diverse Bacterial Partners of Anaerobic Methanotrophic Archaea
Source: mBio. 2017 Aug 1;8(4):e00530-17. doi: 10.1128/mBio.00530-17 (PMC5539420; doi:10.1128/mBio.00530-17)
Supplement: TEXT S1 [file mbo004173410s1.docx]

## Bin description

Eighteen genome bins were recovered from six different sediment samples with the majority being obtained from Hydrate Ridge (Supplementary Table 1). The mean completeness of the bins was 81% (range: 64% - 96%) while contamination averaged 4% (range: 0% - 16%). Four of the genome bins contained 16S rRNA genes which allowed them to be classified into either the SEEP-SRB1 or SEEP-SRB4 clades. The phylogeny of the remaining genome bins could then be inferred from their position in the genome tree. Three of the genome bins belonged to SEEP-SRB4, a small clade found within the *Desulfobulbaceae* (Figure 1). These three bins were highly similar to each other (AAI: 98%) however they were distinct genomes assembled from different Hydrate Ridge sediment samples. SEEP-SRB1 is the largest, most diverse and most frequently observed in methane seeps and has been previously separated into 6 sub-clades (SEEP-SRB1a - SEEP-SRB1f) [(1)](https://paperpile.com/c/RtSLCW/PRSz). A single genome bin, *Desulfobacterales* sp. PC51MH44, contained a 16S rRNA gene classified as SEEP-SRB1a while two genome bins, *Desulfobacterales* sp. S7086C20 and *Desulfobacterales* sp. C00003060, were members of SEEP-SRB1c. In the genome tree the SEEP-SRB1a and SEEP-SRB1c are polyphyletic, with some cultured members of the *Desulfobacterales* separating them (Figure 1). The SEEP-SRB1a and SEEP-SRB1c genomes were diverse, with an average amino acid identity (AAI) of 60% though with some genomes clearly representing closely related strains (AAI: 98%). Surprisingly, the 16S rRNA gene from *Desulfosarcina* sp. BuS5 [(2)](https://paperpile.com/c/RtSLCW/qA4a) was associated with SEEP-SRB1 in both the 16S and genome trees (Figure 1; Figure 16S_TREE). *Desulfosarcina* sp. BuS5 was enriched from a methane seep however it does not appear to be an ANME partner. The position in the 16S and genome trees is supported by AAI analysis that showed that *Desulfosarcina* sp. BuS5 and members of the SEEP-SRB1a are more similar (~62%) compared with other *Desulfosarcina* and *Desulfococcus* (~55%).

## SEEP-SRB4 metabolism

SEEP-SRB4 is a small clade of the *Desulfobulbaceae* that have been identified in a number of methane seeps, but not as ANME partner bacteria [(3)](https://paperpile.com/c/RtSLCW/82j3). Like the SEEP-SRB1, the genomes encoded the Wood-Ljungdahl pathway and the ability to store glycogen using the Embden–Meyerhof–Parnas pathway. However the the SEEP-SRB4 genomes did not contain nitrogenase, but instead contained a periplasmic nitrate reductase. Curiously, no other steps of the denitrification pathway were identified, however this may be due to incompleteness as the three genome bins were estimated to be missing between 20 - 30% of their genomes. Like the SEEP-SRB1 they contained all of the genes for the core sulfate reduction pathway, yet they lacked some of the redox active and membrane complexes observed in SEEP-SRB1 including flx-hdr, Tmc and Qrc. SEEP-SRB4 alternatively contains NADH dehydrogenase for recycling quinones, in contrast to SEEP-SRB1 which contains the Qrc complex. This change could drastically change respiration between the two clades as Qrc accepts electrons from periplasmic sources, whereas NADH dehydrogenase uses cytoplasmically derived NADH to recycle quinones.

The SEEP-SRB4 genome bins also contained an operon with a large multiheme cytochrome with 19 heme binding motifs. Unlike the operons found in the SEEP-SRB1, no beta propeller fold proteins were identified however the surrounding genes contained additional cytochromes, an NrfD-like protein, a trans-membrane di-heme cytochrome and a ferredoxin that would suggest that this cluster is important to electron transfer. The SEEP-SRB4 operon does not appear to be present in the genomes of the other cultured *Deltaproteobacteria* (n=192) but was identified in metagenomes from estuarine sediments [(4)](https://paperpile.com/c/RtSLCW/xaRc6) and from an enrichment culture growing on hydrocarbons [(5)](https://paperpile.com/c/RtSLCW/mwnnI).

##

## References

1. [**Schreiber L**, **Holler T**, **Knittel K**, **Meyerdierks A**, **Amann R**. 2010. Identification of the dominant sulfate-reducing bacterial partner of anaerobic methanotrophs of the ANME-2 clade. Environ Microbiol **12**:2327–2340.](http://paperpile.com/b/RtSLCW/PRSz)

2. [**Kniemeyer O**, **Musat F**, **Sievert SM**, **Knittel K**, **Wilkes H**, **Blumenberg M**, **Michaelis W**, **Classen A**, **Bolm C**, **Joye SB**, **Widdel F**. 2007. Anaerobic oxidation of short-chain hydrocarbons by marine sulphate-reducing bacteria. Nature **449**:898–901.](http://paperpile.com/b/RtSLCW/qA4a)

3. [**Kleindienst S**, **Ramette A**, **Amann R**, **Knittel K**. 2012. Distribution and in situ abundance of sulfate-reducing bacteria in diverse marine hydrocarbon seep sediments. Environ Microbiol **14**:2689–2710.](http://paperpile.com/b/RtSLCW/82j3)

4. [**Baker BJ**, **Lazar CS**, **Teske AP**, **Dick GJ**. 2015. Genomic resolution of linkages in carbon, nitrogen, and sulfur cycling among widespread estuary sediment bacteria. Microbiome **3**:14.](http://paperpile.com/b/RtSLCW/xaRc6)

5. [**Embree M**, **Liu JK**, **Al-Bassam MM**, **Zengler K**. 2015. Networks of energetic and metabolic interactions define dynamics in microbial communities. Proc Natl Acad Sci U S A **112**:15450–15455.](http://paperpile.com/b/RtSLCW/mwnnI)
