# Supplementary material for: Methane-Fueled Syntrophy through Extracellular Electron Transfer: Uncovering the Genomic Traits Conserved within Diverse Bacterial Partners of Anaerobic Methanotrophic Archaea
Source: mBio. 2017 Aug 1;8(4):e00530-17. doi: 10.1128/mBio.00530-17 (PMC5539420; doi:10.1128/mBio.00530-17)
Supplement: FIG S5 [file mbo004173410sf5.ppt]

## Slide 1
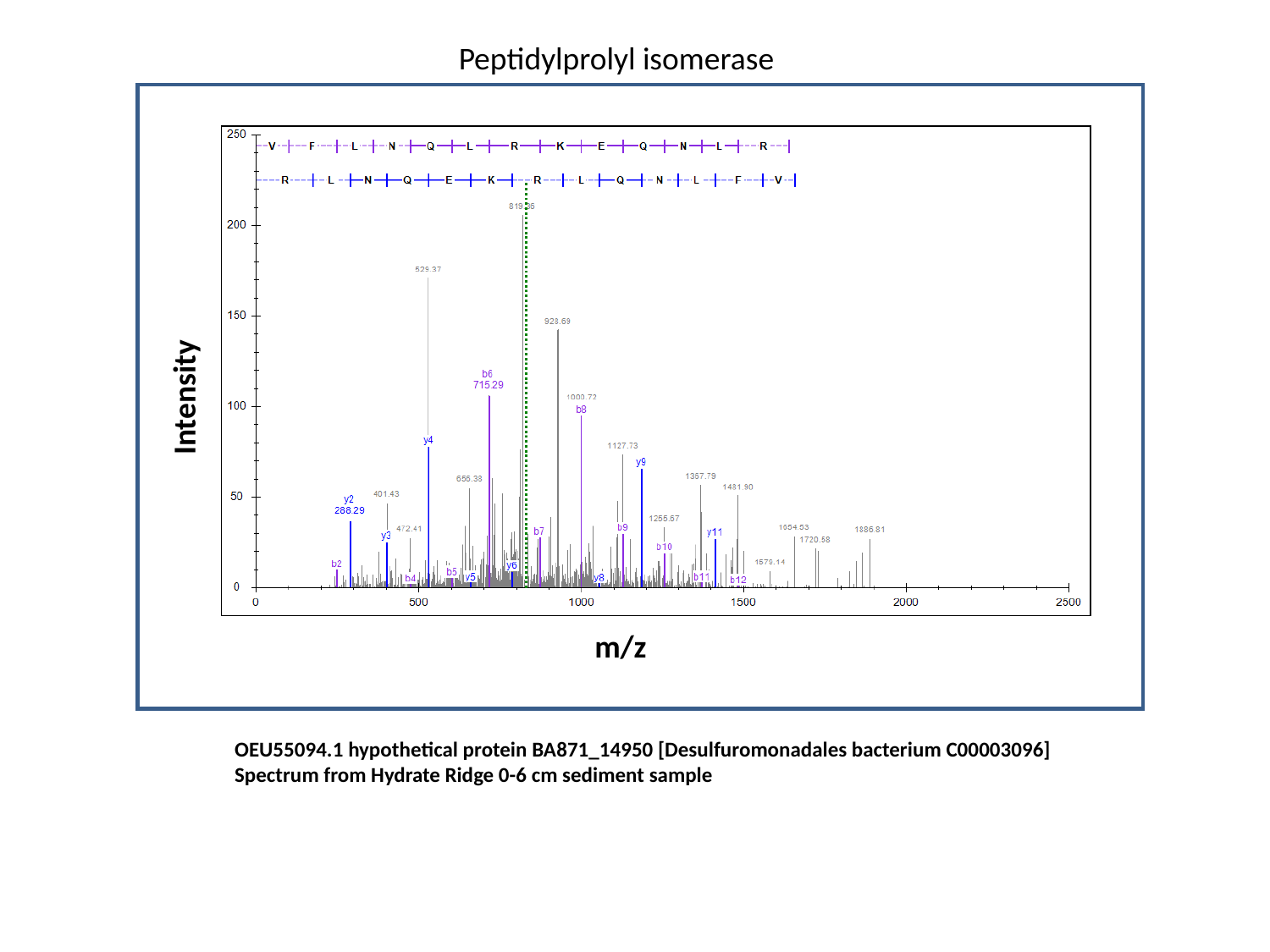

Peptidylprolyl isomerase
Intensity
m/z
OEU55094.1 hypothetical protein BA871_14950 [Desulfuromonadales bacterium C00003096]
Spectrum from Hydrate Ridge 0-6 cm sediment sample

## Slide 2
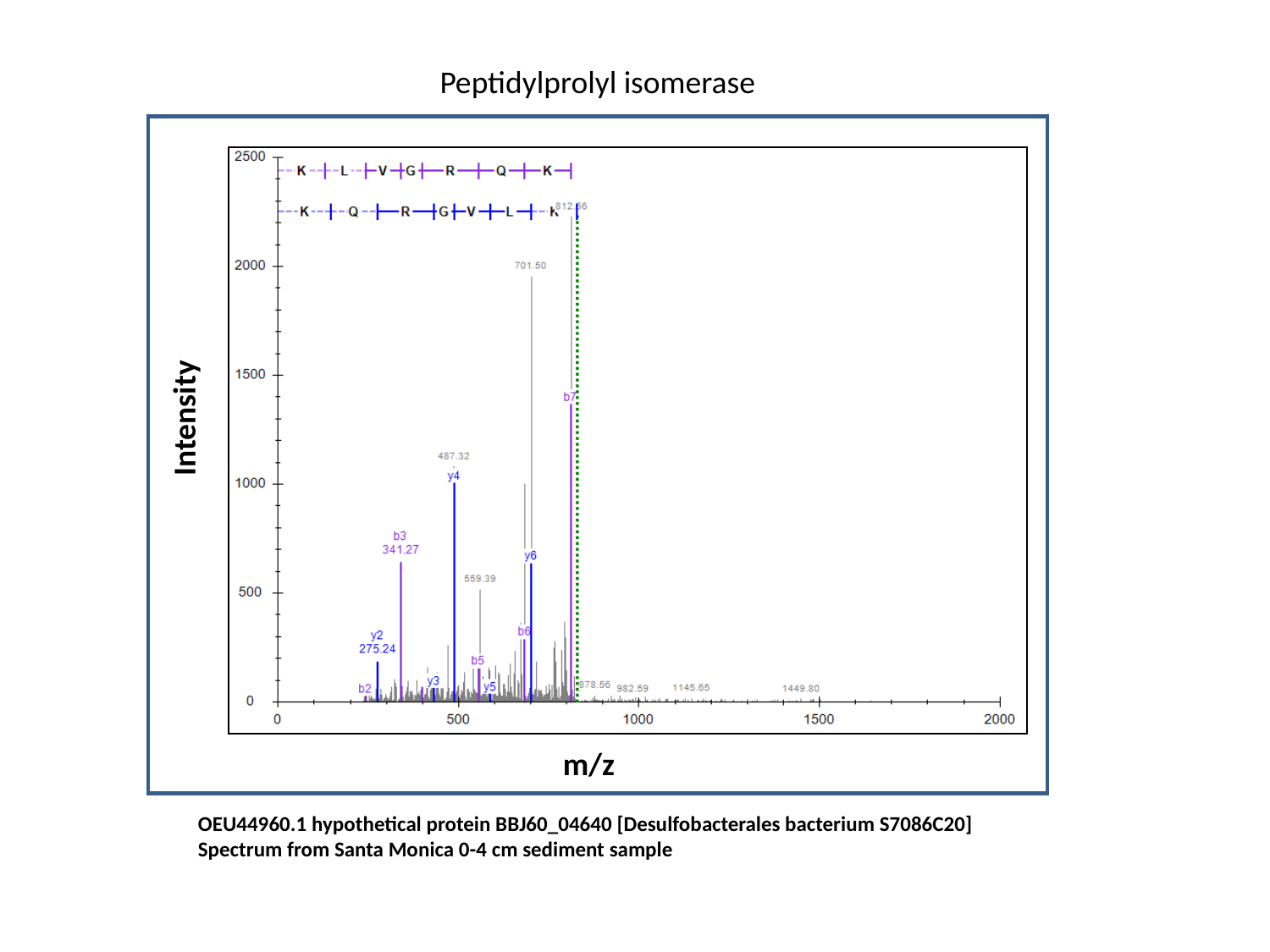

Peptidylprolyl isomerase
Intensity
m/z
OEU44960.1 hypothetical protein BBJ60_04640 [Desulfobacterales bacterium S7086C20]
Spectrum from Santa Monica 0-4 cm sediment sample

## Slide 3
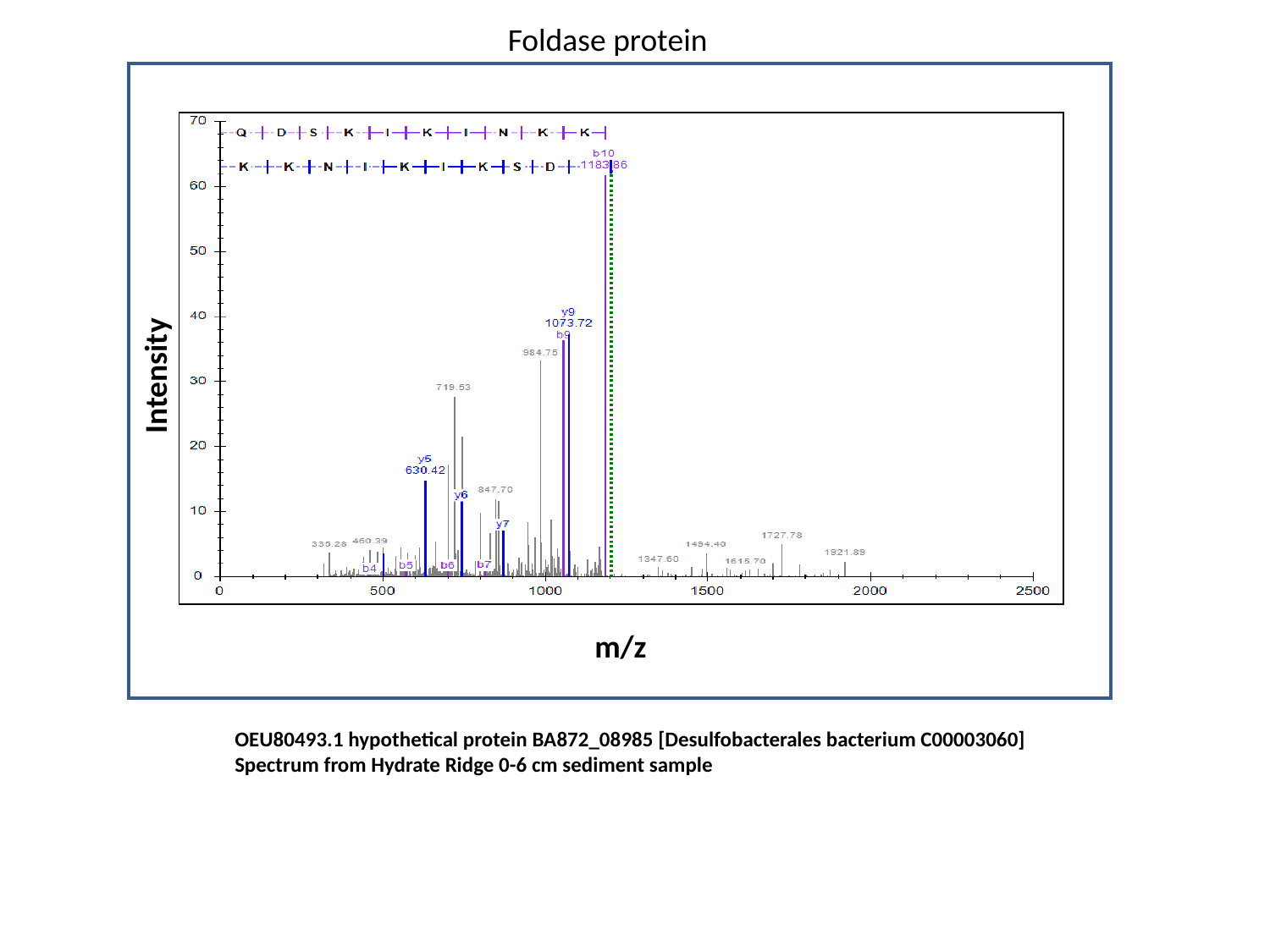

Foldase protein
Intensity
m/z
OEU80493.1 hypothetical protein BA872_08985 [Desulfobacterales bacterium C00003060]
Spectrum from Hydrate Ridge 0-6 cm sediment sample

## Slide 4
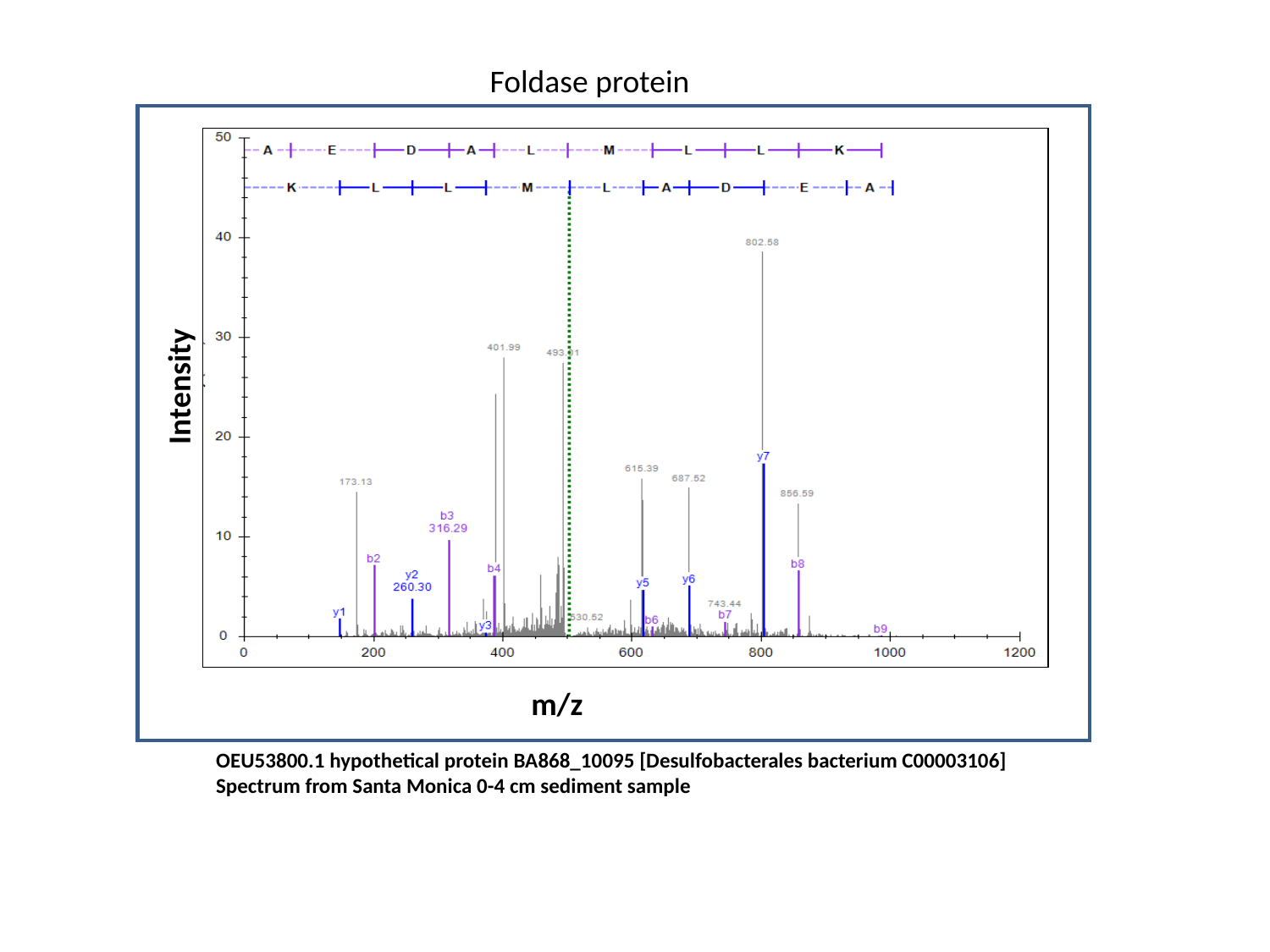

Foldase protein
Intensity
m/z
OEU53800.1 hypothetical protein BA868_10095 [Desulfobacterales bacterium C00003106]
Spectrum from Santa Monica 0-4 cm sediment sample

## Slide 5
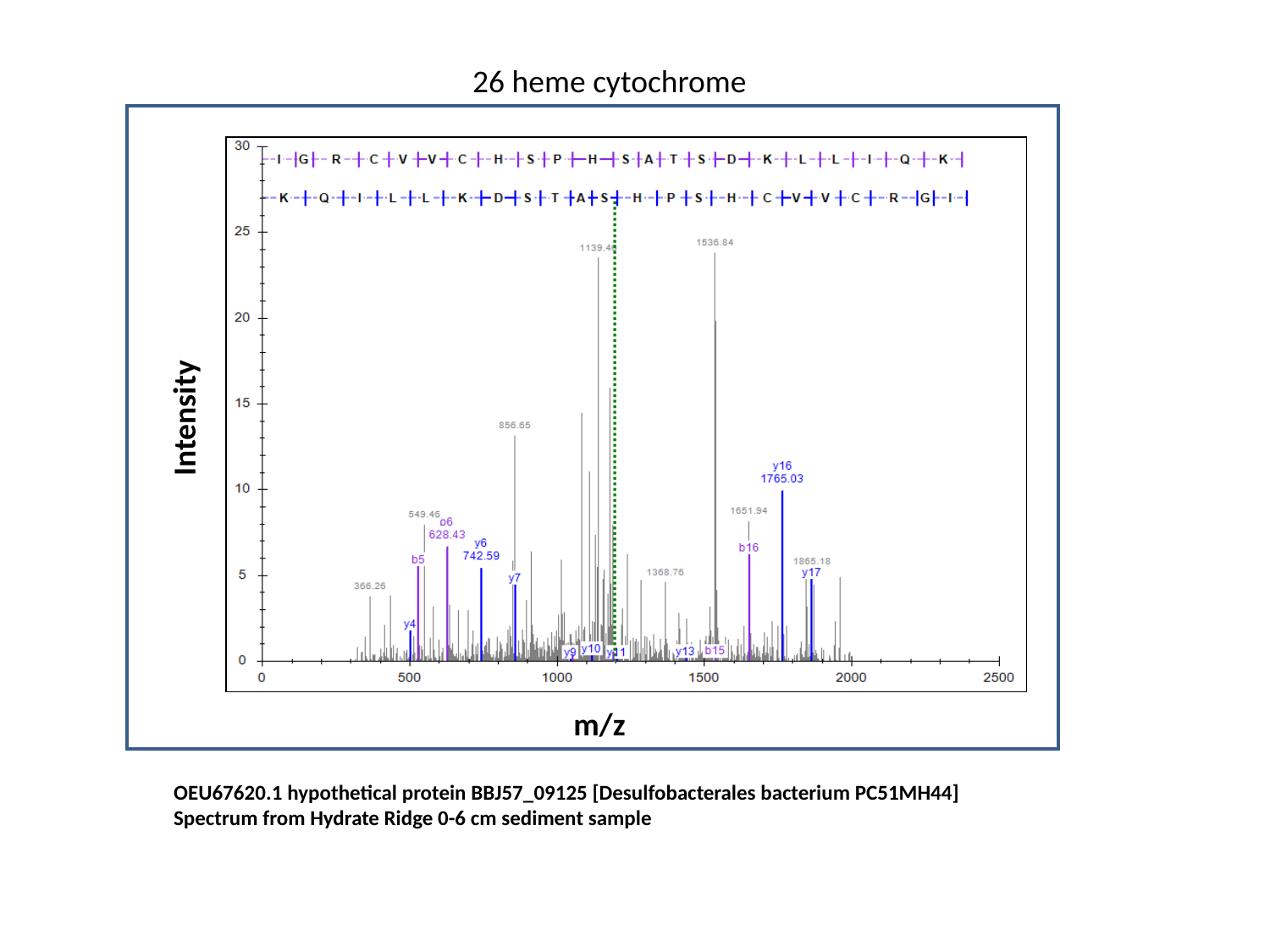

26 heme cytochrome
Intensity
m/z
OEU67620.1 hypothetical protein BBJ57_09125 [Desulfobacterales bacterium PC51MH44]
Spectrum from Hydrate Ridge 0-6 cm sediment sample

## Slide 6
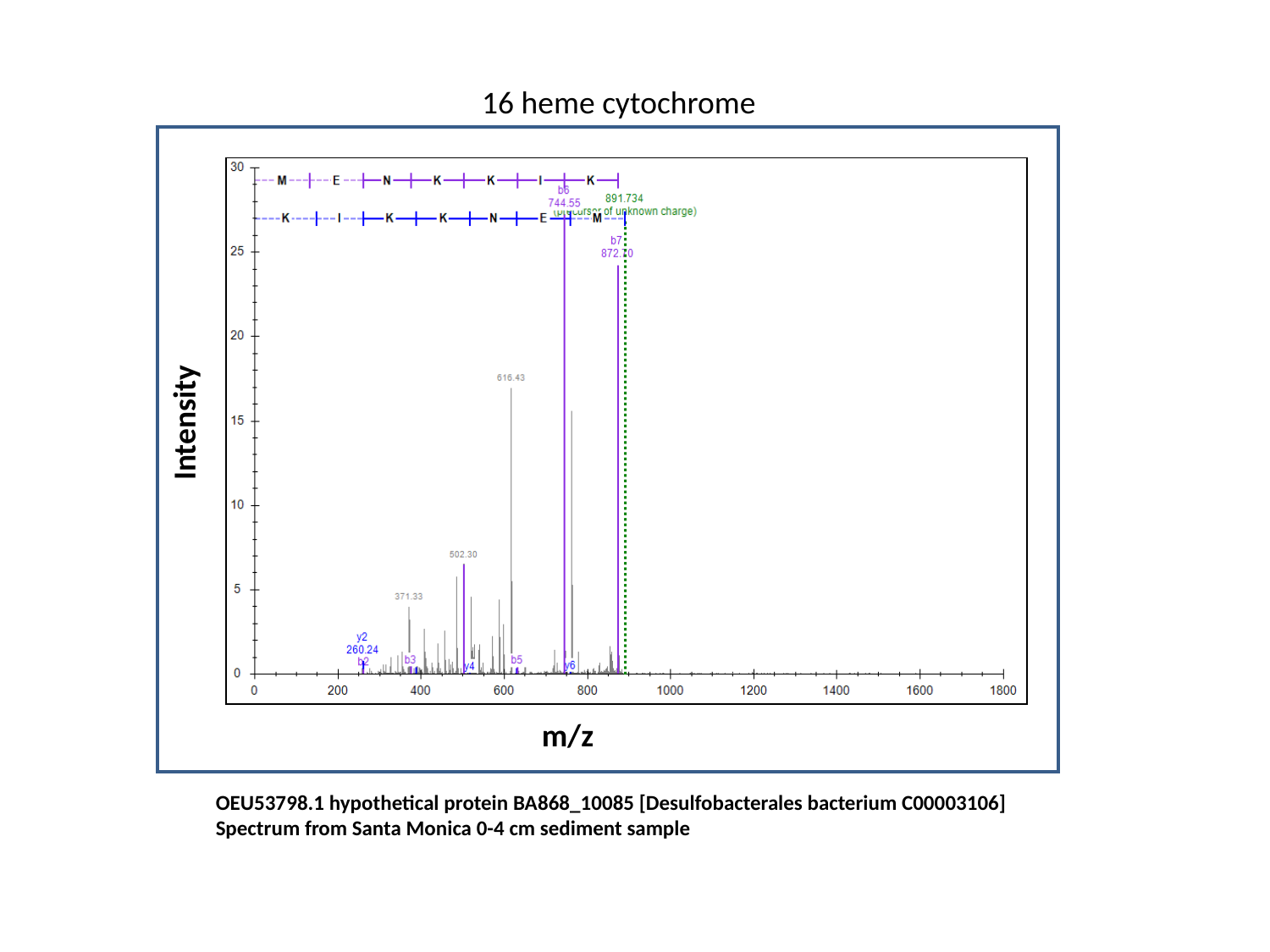

16 heme cytochrome
Intensity
m/z
OEU53798.1 hypothetical protein BA868_10085 [Desulfobacterales bacterium C00003106]
Spectrum from Santa Monica 0-4 cm sediment sample

## Slide 7
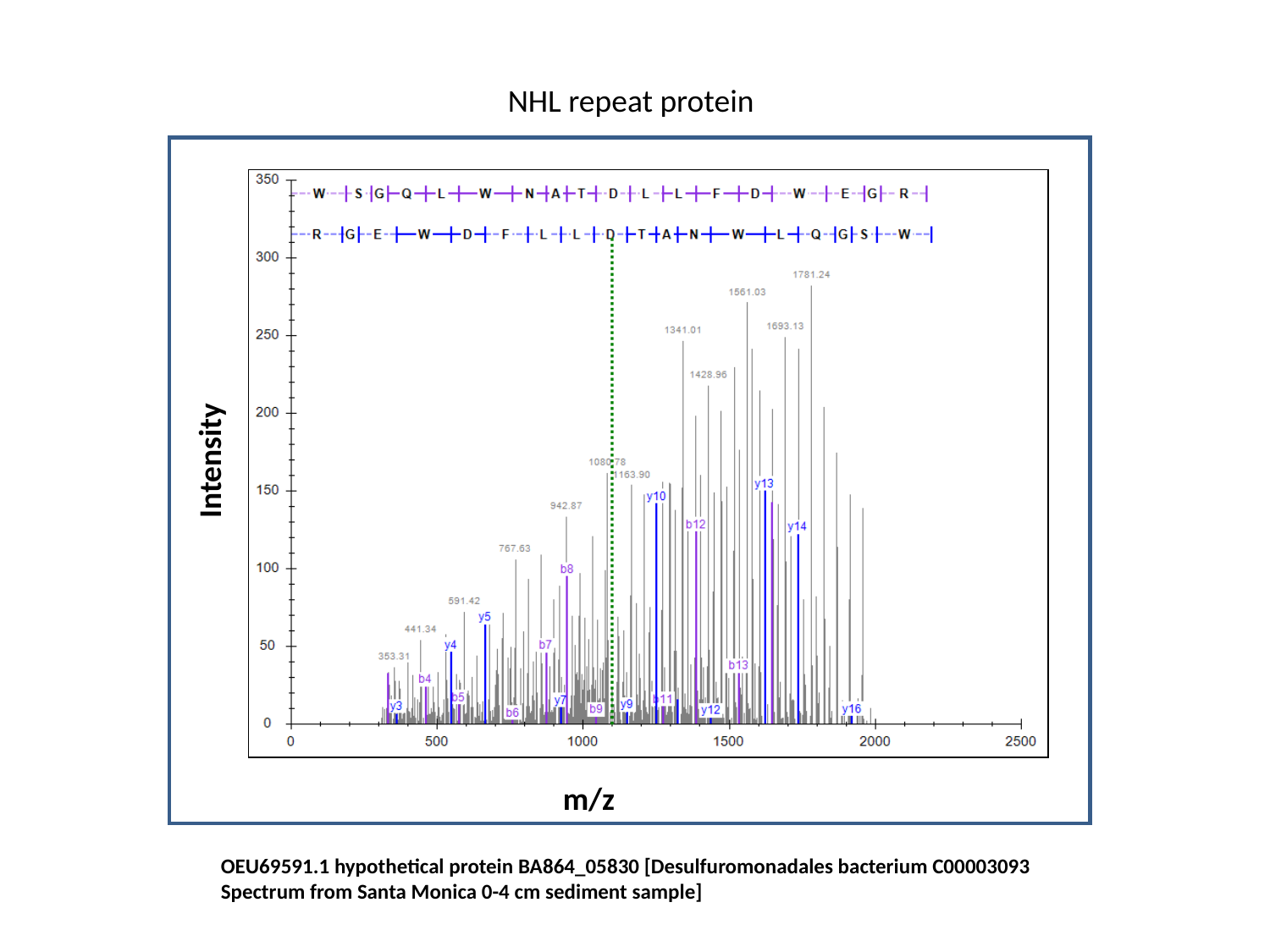

NHL repeat protein
Intensity
m/z
OEU69591.1 hypothetical protein BA864_05830 [Desulfuromonadales bacterium C00003093
Spectrum from Santa Monica 0-4 cm sediment sample]
